# Supplementary material for: Deciphering the Molecular Mechanisms of Autonomic Nervous System Neuron Induction through Integrative Bioinformatics Analysis
Source: Int J Mol Sci. 2023 May 21;24(10):9053. doi: 10.3390/ijms24109053 (PMC10219521; doi:10.3390/ijms24109053)
Supplement: Supplementary file 1 [file ijms-24-09053-s001.zip › Caption.pdf]

Table S1: Enriched terms of the upregulated DEGs in day 13 compared to day 0; Table S2: Enriched terms of the downregulated DEGs in day 13 compared to day 0; Table S3: Enriched terms of genes in each cluster identified by MCODE plugin in day 7 compared to day 0; Table S4: Enriched terms of genes in each cluster identified by MCODE plugin in day 13 compared to day 7; Video S1: Pharmacological evaluation of induced neurons with L-glutamate and nicotine at day 33; Video S2: Pharmacological evaluation of induced neurons with capsaicin and menthol at day 33.
